# Repeated biliary metal stent fracture and migration in malignant biliary obstruction

**DOI:** 10.1055/a-2934-6755

**Published:** 2026-08-19

**Authors:** Kei Yane, Daichi Watanabe, Yuki Ikeda

**Affiliations:** 1 Department of Gastroenterology 36737 Tonan HospitalSapporoHokkaidoJapan

A 63-year-old man after distal gastrectomy with Roux-en-Y reconstruction for gastric cancer was receiving chemotherapy for peritoneal dissemination. He developed a distal biliary stricture, and double-balloon enteroscopy-assisted endoscopic retrograde cholangiopancreatography (ERCP) was performed. Biliary biopsy revealed poorly differentiated adenocarcinoma, indicating malignant biliary obstruction caused by the peritoneal dissemination of gastric cancer.

**Fig. 1:**
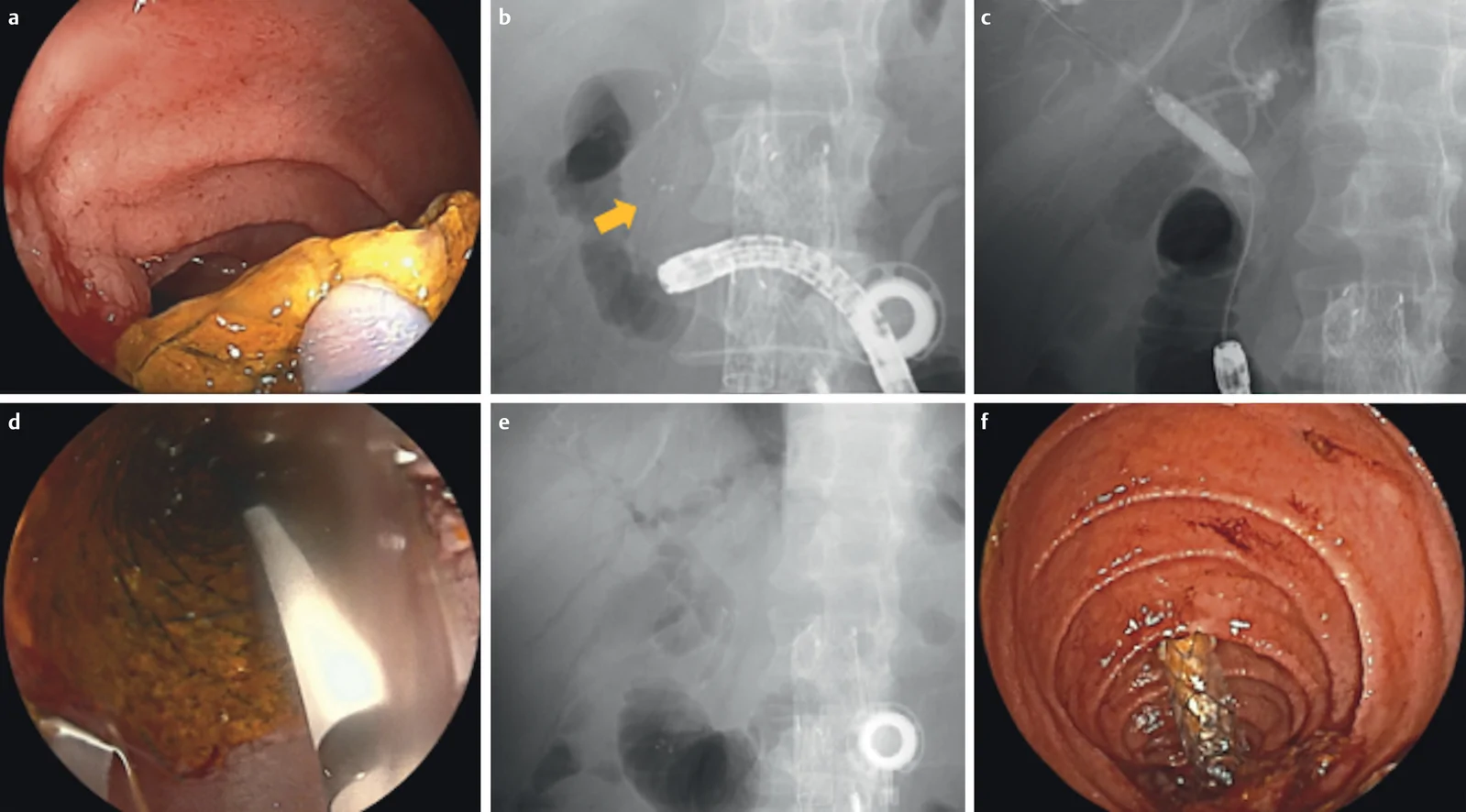
Endoscopic removal of the fractured and migrated SEMS. ( **a** ) During emergency ERCP, attempted removal with a snare caused SEMS fracture, and the fractured fragment migrated proximally into the bile duct. ( **b** ) A fluoroscopic image showing the fractured stent (arrow). ( **c** ) A guidewire was advanced through the migrated stent, and an 8-mm EPBD balloon was inflated within the stent lumen. ( **d** ) The fractured stent fragment was removed by pulling the inflated EPBD balloon.( **e** ) A fluoroscopic image after SEMS replacement. ( **f** ) An endoscopic image after SEMS replacement.

One month after plastic stent placement, cholangitis occurred, and the stent was replaced with a fully covered self-expandable metal stent (SEMS; HANAROSTENT Biliary Full Cover Benefit, 6 mm×60 mm, Boston Scientific, MA, USA). Six months later, severe cholangitis recurred. During emergency ERCP, attempted stent removal with a snare caused SEMS fracture, and the fractured fragment migrated proximally into the bile duct ( Fig. 1a, b ). A guidewire was advanced through the migrated stent, and the fragment was removed by inflating an 8-mm endoscopic papillary balloon dilation (EPBD) balloon (REN biliary dilation catheter; KANEKA, Osaka, Japan) within the stent lumen ( Fig. 1c, d ). The SEMS was replaced with the same type of fully covered SEMS ( Fig. 1e, f ), and cholangitis improved.

**Fig. 2:**
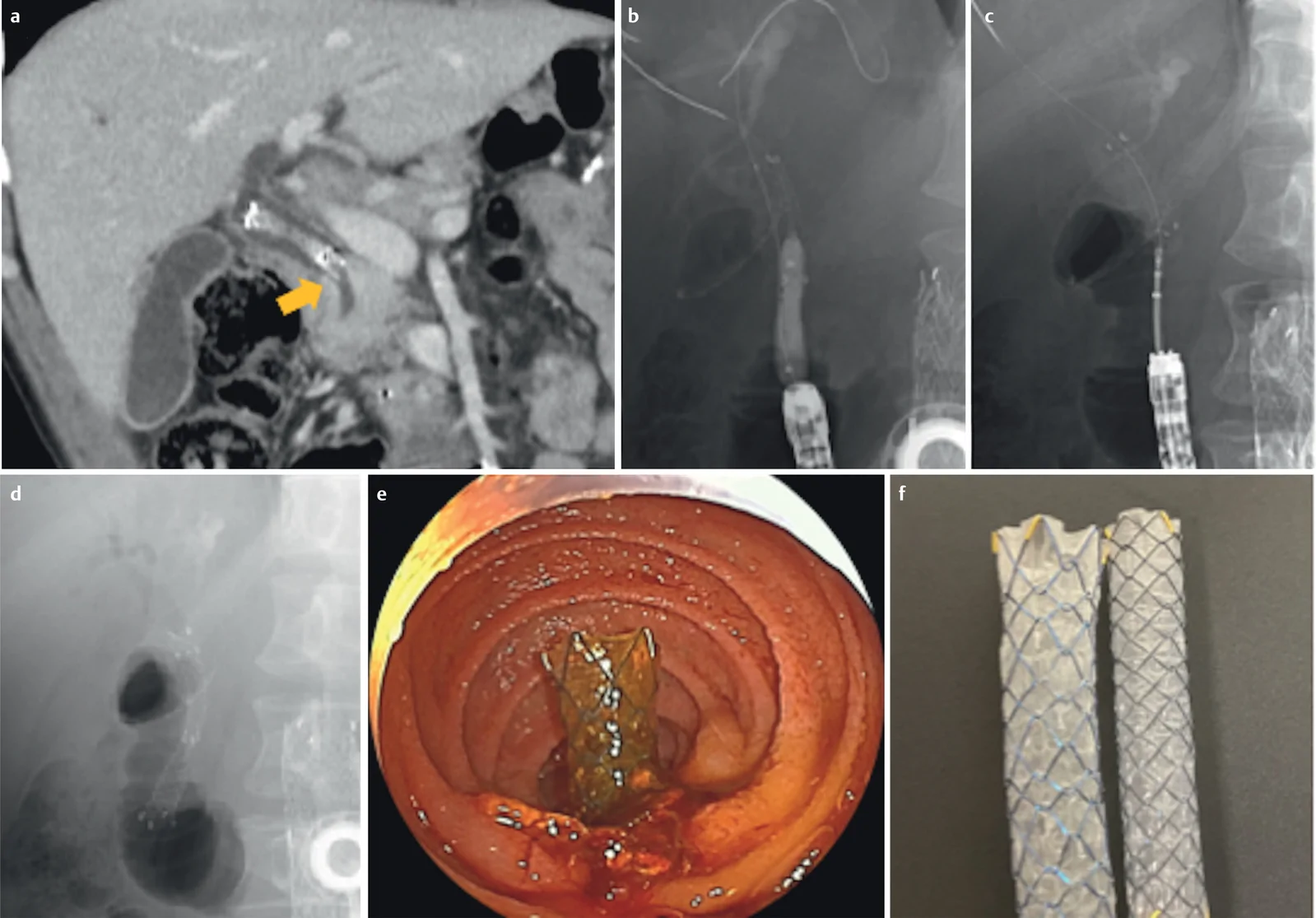
Management of recurrent SEMS fracture and migration. ( **a** ) Computed tomography showed the spontaneous fracture of the SEMS with proximal migration of the fractured fragment (arrow). ( **b** ) EPBD balloon-assisted SEMS removal failed because of severe biliary stricture. ( **c** ) SEMS removal using a device delivery system and biopsy forceps was also unsuccessful. ( **d** ) A fluoroscopic image after additional SEMS placement. ( **e** ) An endoscopic image after additional SEMS placement. ( **f** ) HANAROSTENT biliary NEO (left, wire diameter: 0.65 mm) and HANAROSTENT biliary benefit (right, wire diameter: 0.43 mm).

Five months later, cholangitis recurred. Computed tomography showed the spontaneous fracture of the SEMS with proximal fragment migration ( Fig. 2a ). Although EPBD balloon-assisted removal was attempted, extraction failed because the proximal end was impacted at the stricture ( Fig. 2b ). Removal using a device delivery system (EndoSheather, Piolax, Kanagawa, Japan) and biopsy forceps also failed ( Fig. 2c ). Therefore, an additional metal stent (HANAROSTENT Biliary Multi-hole NEO, 8 mm×60 mm, Boston Scientific) was deployed inside the fractured stent remnant ( Fig. 2d, e ). Cholangitis improved, with no stent dysfunction for 6 months ( Video 1 ).

**Video 1** Endoscopic management of repeated fracture and migration of a biliary metal stent in malignant biliary obstruction caused by peritoneal dissemination of gastric cancer. Video URI:


Although spontaneous SEMS fracture in malignant biliary obstruction has been reported, 1​ 2​ 3​ ​4​ repeated fracture and migration are rare. Severe stricture and thin-wire stent design may have contributed to repeated fracture ( Fig. 2f ). This case highlights the importance of appropriate management for fractured and migrated biliary metal stents.

Endoscopy_UCTN_Code_CPL_1AK_2AD
